# Modulating Vesicle Priming Reveals that Vesicle Immobilization Is Necessary but not Sufficient for Fusion-Competence

**DOI:** 10.1371/journal.pone.0002694

**Published:** 2008-07-16

**Authors:** Ofer Yizhar, Uri Ashery

**Affiliations:** Department of Neurobiology, Faculty of Life Sciences, Tel Aviv University, Tel Aviv, Israel; University of California, Berkeley, United States of America

## Abstract

In neurons and neuroendocrine cells, docked vesicles need to undergo priming to become fusion competent. Priming is a multi-step process that was shown to be associated with vesicle immobilization. However, it is not known whether vesicle immobilization is sufficient to acquire complete fusion competence. To extend our understanding of the physical manifestation of vesicle priming, we took advantage of tomosyn, a SNARE-related protein that specifically inhibits vesicle priming, and measured its effect on vesicle dynamics in live chromaffin cells using total internal reflection fluorescence microscopy. We show here that while in control cells vesicles undergo immobilization before fusion, vesicle immobilization is attenuated in tomosyn overexpressing cells. This in turn increases the turnover rate of vesicles near the membrane and attenuates the fusion of newcomer vesicles. Moreover, the release probability of immobile vesicles in tomosyn cells is significantly reduced, suggesting that immobilization is an early and necessary step in priming but is insufficient, as further molecular processes are needed to acquire complete fusion competence. Using tomosyn as a molecular tool we provide a mechanistic link between functional docking and priming and suggest that functional docking is the first step in vesicle priming, followed by molecular modifications that do not translate into changes in vesicle mobility.

## Introduction

In neurons and neuroendocrine cells, vesicles translocate from the cytoplasm to the plasma membrane to undergo a molecular process called priming that renders them fusion-competent [1]. Primed vesicles, which constitute the readily releasable pool of vesicles (RRP), can then be rapidly exocytosed in response to elevation in intracellular calcium, giving rise to the rapid initial kinetic component of exocytosis [2]–[6]. Under prolonged stimulation, this phase is followed by the fusion of vesicles that have undergone priming during stimulation, giving rise to a slower kinetic phase [7]–[9]. Previous work has shown that the existence of this readily releasable (primed) pool of vesicles requires formation of the SNARE (soluble NSF-attachment protein receptor) complex—a heterotrimeric complex composed of Syntaxin and SNAP25 on the plasma membrane and VAMP/Synaptobrevin on the vesicle membrane [10]–[13]. In the last decade, multiple proteins have been identified as priming regulators 1, 14. Among the most prominent priming factors are Munc13, which increases the size of the RRP in chromaffin cells and is nessesary for synaptic transmission in neurons [7], [15]–[17] and tomosyn, which selectively reduces the size of the RRP in chromaffin cells [18] and inhibits synaptic transmission in neurons [19]–[21].

Tomosyn is a 130-kD cytoplasmic protein that was identified as a binding partner of Syntaxin1A [22]. Tomosy contains an R-SNARE coiled-coil domain in its C-terminus, separated with a hypervariable domain from the N-terminal WD40-repeat domain. The tomosyn WD40 domain and the adjacent hypervariable domain are predicted to fold into a twin-beta propeller structure that can serve as a platform for protein-protein interactions or regulate the activity of the SNARE domain through intra-protein interactions [23]. These domains serve as a minimal functional domain [24] but tomosyn's activity is regulated by the interaction of its SNARE motif with Syntaxin [25]. Tomosyn overexpression has been shown to inhibit exocytosis in PC12 cells, chromaffin cells, insulin-secreting cells, adipocytes and neurons [18], [19], [26]–[28]. In addition, tomosyn was shown to localize to the palms of extending neuronal growth cones and its overexpression inhibited neurite outgrowth in hippocampal neurons. It was therefore proposed that by preventing the fusion of vesicles at the growth-cone palm through its interaction with syntaxin, tomosyn directs the vesicles to fuse at the leading edge of the elongating growth cone [29]. These phenomena may all be related to an inability to maintain a fusion-competent pool of vesicles under conditions in which tomosyn is abundant and to the inhibition of vesicle priming that occurs when tomosyn is overexpressed [18], [30].

Despite significant progress in our understanding of the biochemistry and physiology of vesicle priming [1], much less is known about the physical manifestation of the primed state and the processes that take place between the vesicle's arrival at the membrane and its subsequent fusion. In earlier electrophysiological studies, vesicle docking and priming were indirectly assessed by quantifying the kinetics of exocytosis in response to stimulation and measuring the distance of vesicles from the plasma membrane in fixed cells using electron microscopy [7], [31]. Recently, total internal reflection fluorescence microscopy (TIRFM) has been used to observe vesicles in live cells [32]–[36] and the events preceding vesicle fusion are starting to become clear. Vesicle docking was shown to correspond with changes in the axial mobility and resident time of vesicles near the membrane [37], while priming was suggested to be associated with restricted lateral mobility [38]. Nevertheless, several recent studies have demonstrated that interfering with proteins known to be involved in priming and fusion significantly alters vesicle docking [30], [39], [40], suggesting that vesicle docking and priming are interlinked. To date, it is not known whether vesicle immobilization is sufficient to attain the primed state, or whether it constitutes a preliminary step in this process. It is also of interest to examine what are the dynamic effects on the equilibrium between vesicle docking and priming upon inhibition of vesicle priming. Thus, the aim of this study was to examine whether the profound effect of tomosyn on vesicle fusion is related to changes in vesicle dynamics near the plasma membrane in live cells, under both resting and stimulated conditions. We show that under resting conditions, tomosyn overexpression inhibits the immobilization of vesicles that arrive at the plasma-membrane region and enhances the turnover of membrane-proximal vesicles. Upon stimulation, tomosyn-overexpressing cells secrete with slower kinetics owing to inhibition of the fusion of resident vesicles and to a significant slowing in the immobilization and fusion of newcomer vesicles.

## Results

### Tracking and mobility analysis of young chromaffin vesicles

**Figure 1:**
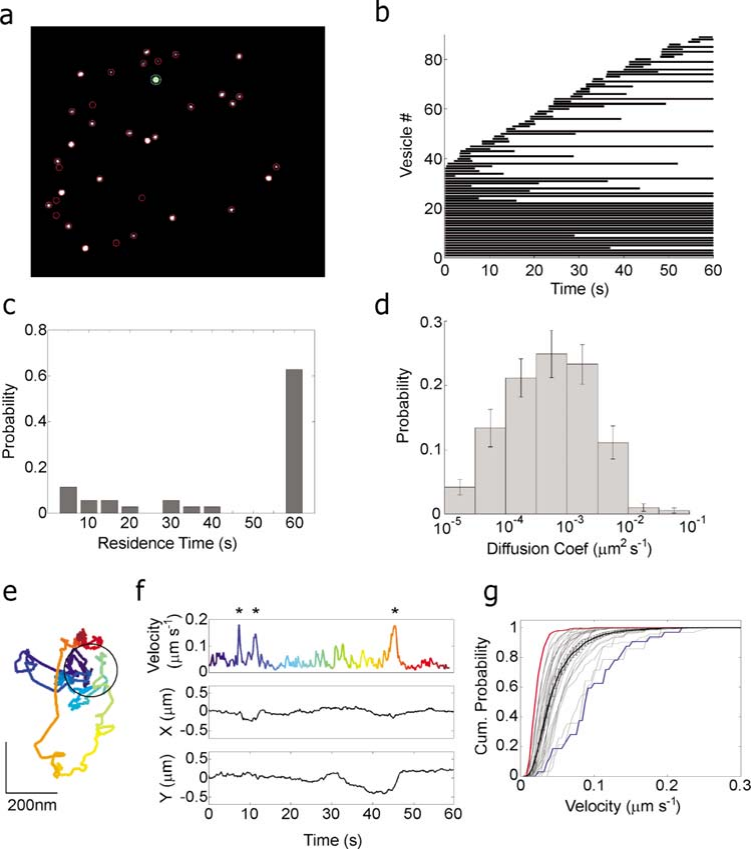
Tracking and mobility analysis of vesicles in a representative cell. (a). First image in a time-lapse image sequence of a representative control cell expressing NPY-Venus. Vesicles (circled) were identified by their fluorescence profile and tracked throughout the sequence. Vesicles that appeared later in the sequence were also identified and tracked. (b). A kymograph representing the duration of vesicle residence near the membrane. Individual vesicles are represented as lines initiating at vesicle appearance and terminating at vesicle disappearance from the TIRF plane (vesicles 1–39 were present at the start of the time-lapse sequence). (c). Histogram depicting the lifetime distribution of vesicles in the representative cell shown in a. (d). Average logarithmically binned histogram showing the distribution of vesicle diffusion coefficients in control cells (n = 18 cells). (e). Time-coded trajectory for the vesicle marked with a green circle in a. The vesicle was tracked for 60 s (see f for color code). Black circle indicates the size of an average chromaffin vesicle. (f). For the same vesicle shown in e, X and Y coordinates are shown separately (middle and bottom traces, respectively) with a windowed-velocity graph (top) that is color-coded as in e. Asterisks denote periods of high mobility. (g). Normalized cumulative-velocity histograms of all single vesicles in one cell (gray curves) and the mean histogram for the same cell (black; error bars represent SEM). Blue and red histograms describe the mobility of two representative vesicles with high and low mobility, respectively.

To obtain a baseline for the effects of tomosyn overexpression on vesicle mobility, residence and fusion, we first characterized these parameters in control cells. Previous work has shown that newly synthesized vesicles are the first to undergo exocytosis in bovine chromaffin cells [41]. We therefore selectively labeled “young” vesicles by infecting the cells for 8 to 12 h with a virus expressing neuropeptide-Y (NPY) fused to the fluorescent protein Venus [42]. We tracked individual vesicles in live bovine chromaffin cells using a custom-written algorithm (see Methods). In each cell, all visible vesicles were identified based on their typical fluorescence-intensity (FI) distribution (Fig. 1a and Figure S1) and tracked for the entire duration of time-lapse acquisition. Figure 1b shows a kymograph representation of vesicle lifetimes in a single resting cell, in which each vesicle is represented by a line extending from the time of vesicle appearance (docking) to its disappearance (retraction into the cytoplasm). Some vesicles persisted in the TIRF plane throughout the acquisition period, while others appeared for shorter periods and then disappeared (Fig. 1b). We were interested in two fundamental aspects of vesicle behavior: (1) the duration of residence of each vesicle near the plasma membrane, and (2) their mobility under different conditions. We observed that a significant proportion of the “new” vesicles were actually vesicles that disappeared and reappeared in the TIRF plane within several acquisition frames, such that the tracking algorithm could not automatically identify them as the same vesicle. This would introduce a significant bias into any statistical analysis of vesicle mobility, as these vesicles would be over-represented according to the number of times they reappear at the membrane. Taking this into account, we measured the residence time at the plasma membrane of only those vesicles that were present at *t* = *0* (Fig. 1b, vesicles 1–39), and disregarded those that arrived later. Measuring vesicle residence time at the membrane demonstrated that there are two populations of vesicles: 40% of the vesicles sample the membrane, remaining within the TIRF region for a short duration (2–40 sec) and a second population of vesicles resides at the membrane for longer periods of times (over 60 seconds; Fig. 1c). We refer to the short-lived vesicles as “newcomer” vesicles and to the long-lived ones as “resident” vesicles.

We then quantified the lateral mobility of vesicles in resting cells by calculating the apparent diffusion coefficient for each vesicle (Fig. 1d) according to [43]–[45]. The results are consistent with previous reports and indicate that the mean mobility of chromaffin vesicles is widely distributed across several orders of magnitude. We further observed that the mobility of single vesicles is extremely nonuniform (Fig. 1e) and can vary within a short time interval. For instance, the mobility of the vesicle shown in Figure 1e varied greatly during the 60 s of tracking, a situation that would not be reflected by a global calculation of diffusion coefficient. We therefore devised an improved representation of vesicle mobility, which involves calculating the point-to-point velocity (ptpv) at each point in the time series. This is a sensitive measure of the mobility behavior of a vesicle and can accurately detect the rapid transitions from immobility to enhanced mobility that are often observed with these vesicles (Fig. 1f, asterisks). To represent the entire range of movements displayed by each vesicle, we then calculated a cumulative distribution function (cdf) that describes their mobilities (Fig. 1g, gray curves). These traces are averaged across vesicles in each cell to yield the representative cdf for the specific cell (Fig. 1g, black curve) and can then be averaged between cells and compared between different experimental conditions (see Analysis S1 for detailed explanation). For example, in the cell depicted in Figure 1, the average cdf curve shows that on average, 90% of the vesicles' movements were smaller than 0.1 µm s^−1^ (Fig. 1g, black curve). To correct for apparent mobility resulting from instrument noise and to measure the minimal mobility that can be detected with our system, we calculated the mobility cdf of immobilized 220-nm-diameter fluorescent beads at varying fluorescence intensities and corrected each vesicle's cdf according to its FI [32], see Figure S3).

### Fusing vesicles in control cells are mostly derived from the resident pool

**Figure 2:**
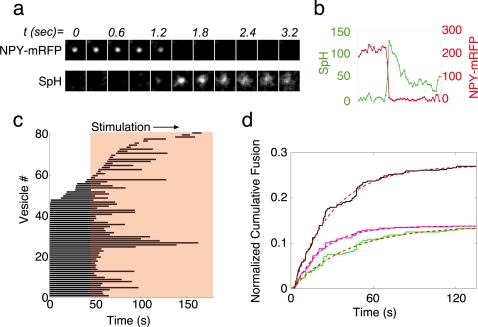
Monitoring fusion events with a combination of NPY-mRFP and synaptopHluorin. (a). Fusion of a single vesicle imaged with dual-wavelength image-splitter. synaptopHluorin (SpH) fluorescence appears at t = 1.2 s, and NPY-mRFP fluorescence disappears at the same time. (b). Fluorescence intensity measured at the site of fusion at both wavelengths. (c). Lifetime plot of vesicles that fused in response to stimulation. Data are pooled from n = 10 cells. Each trace starts at the appearance of a vesicle in the TIRF plane (vesicles 1–45 were present at the start of the time-lapse sequence) and ends with the exocytotic event. (d). Normalized time course of secretion, in which fusion events were accumulated and normalized to the number of vesicles visible in each cell before stimulation. Black trace shows the fusion kinetics of all vesicles. Green and magenta traces show the fusion kinetics of resident and newcomer vesicles, respectively. Each trace is fitted with a single exponential (red dotted lines, see Table 2).

To characterize the vesicle populations that undergo fusion in chromaffin cells, we tracked vesicles in stimulated cells in the period immediately preceding their fusion. Vesicle fusion was positively identified by imaging cells that were co-transfected with NPY-mRFP and superecliptic synaptopHluorin (SpH). SpH is a chimeric protein composed of the transmembrane segment of the vesicle SNARE (v-SNARE) protein Synaptobrevin2/VAMP and pHluorin, the pH-sensitive derivative of GFP [46]. This protein is completely invisible (eclipsed) when pHluorin is inside the acidic lumen of the vesicle. However, when the vesicle is exocytosed, pHluorin is exposed to the neutral pH of the extracellular solution and becomes brightly fluorescent. The cells were imaged simultaneously using TIRFM at the two wavelengths for acquisition of both fluorophores (Fig. 2a), and fusion events were readily identified by the typical rapid disappearance of NPY-mRFP fluorescence concomitant with the appearance of SpH fluorescence that then diffused laterally into the membrane and disappeared (Fig. 2a,b). The stimulation protocol involved imaging the cells for 45 s before stimulation and then applying a solution containing 60 mM KCl to depolarize the cells and induce calcium entry through voltage-gated channels (Fig. 2c).

Analysis of the fusion of 82 vesicles from 10 cells depicted in Fig. 2c showed that most of the vesicles that fused in response to stimulation (71.6%) originated from the resident pool (residence time >30 s), while a smaller percentage (28.4%) of the fusion events originated from vesicles that appeared in the TIRF plane during depolarization (newcomers). A cumulative representation of fusion-event occurrence showed that secretion under these conditions follows exponential kinetics that are typical for this type of cell [47] (Fig. 2d, black curve). We then separated the fusion events into those resulting from residents and those resulting from newcomer vesicles. Exponential fitting of these curves revealed that the resident vesicles fused with faster kinetics (τ = 22.9 s, r^2^ = 0.994) than newcomers (τ = 65.3 s, r^2^ = 0.998) (Fig. 2d, magenta and green traces, respectively). These results are in agreement with previous work on chromaffin cells [48] and indicate that resident vesicles contribute to the fast initial phase of exocytosis, while the arrival of newcomers dictates the rate of the slower phase observed during prolonged stimulation.

### Tomosyn reduces vesicle residence time at the membrane and increases vesicle mobility

**Figure 3:**
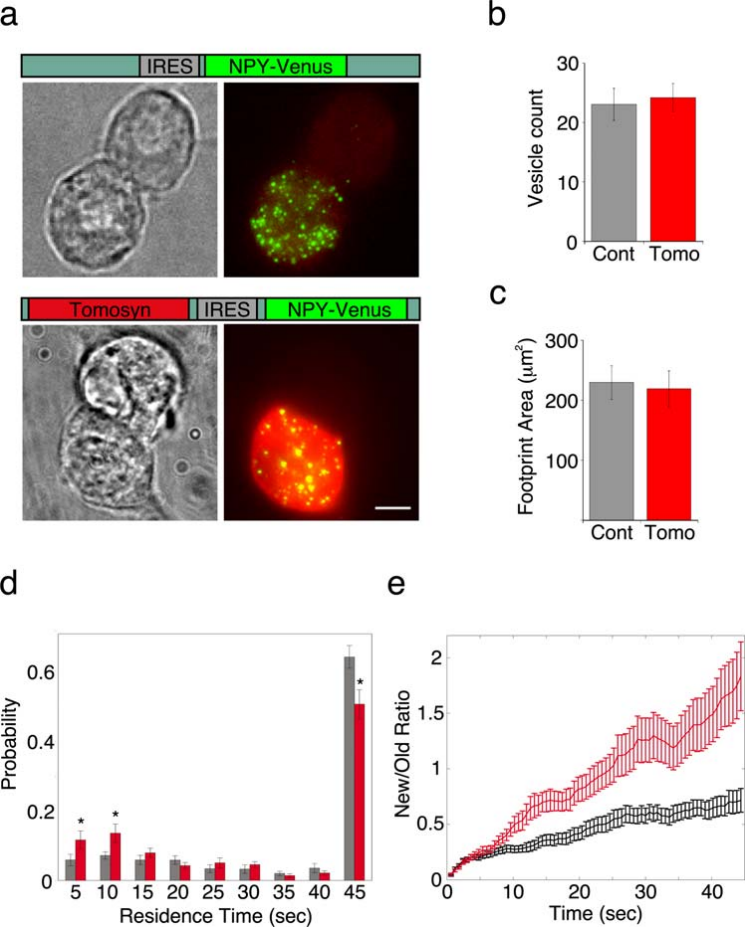
Vesicle residence time at the membrane is reduced and vesicle turnover is enhanced in tomosyn-overexpressing cells. (a). Viral constructs used to express NPY-Venus alone (top) or together with tomosyn (bottom). Cells infected with each virus were fixed and immunolabeled with anti-tomosyn Ab. Shown are phase-contrast images (left) and dual-wavelength fluorescence images of NPY-Venus (green) and tomosyn Ab (red). Infection with tomosyn virus did not change the number of vesicles present near the membrane (b) or the surface area of the cells' footprints on the coverslip (c). (d) Histograms of vesicle residence time in control (black) and tomosyn-overexpressing (red) * p<0.05 (student's t-test). (e). Average ratio between new and old vesicles for each time point in control (black) and tomosyn-overexpressing (red) cells. Statistically significant change (*p*<0.01, Mann-Whitney's rank-sum test) was observed from t = 14 s. All presented data are from 18 control and 25 tomosyn-overexpressing cells.

Previous studies have characterized tomosyn as a potent inhibitor of vesicle priming [18], [21], [30], [49], but the mechanism by which tomosyn exerts these effects remained unexplained. It was therefore of interest to examine the effect of tomosyn overexpression on the mobility of vesicles in bovine chromaffin cells. To image vesicles in tomosyn-overexpressing cells, we used the SFV expression system to express tomosyn and NPY-Venus, separated by an internal ribosomal entry site (IRES) element. Control cells were infected with a virus encoding IRES-NPY-Venus (Fig. 3a). Tomosyn overexpression was confirmed by immunofluorescence analysis with anti-tomosyn antibody (Fig. 3a). Tomosyn overexpression did not cause a significant change in the number of vesicles near the plasma membrane (Fig. 3b; 23±2.7 vs. 24±2.4 vesicles in control and tomosyn-overexpressing cells, respectively) or in the surface area of the cell's footprint on the glass coverslip (Fig. 3c; 229±28 vs. 219±30 µm^2^ in control and tomosyn-overexpressing cells, respectively), but slightly increased the amount of vesicles per membrane area (0.11±0.011 vs. 0.15±0.017 vesicles/µm^2^ in control and tomosyn-overexpressing cells, respectively). This is consistent with previous electron-microscopy results, which showed that the distribution of vesicles in chromaffin cells overexpressing tomosyn does not significantly differ from controls [18].

We first determined the effect of tomosyn on the residence time of vesicles near the membrane. For each cell, we calculated a residence-time histogram such as the one shown in Figure 1c and averaged these histograms across cells in the control and tomosyn-overexpressing groups. The averaged histograms showed that tomosyn causes a significant decrease in the amount of vesicles in the resident pool and a concomitant increase in the amount of newcomers (Fig. 3d). Since we speculated that this situation might reflect a higher turnover rate of vesicles in the membrane region, we performed the following measurement: in each cell, after tracking all of the vesicles visible TIRF for the entire duration of the movie, we marked those vesicles that were present at *t* = *0* (the first image in the sequence) as “old” and any vesicle that appeared during acquisition as “new”. The ratio between new and old vesicles was calculated for each time point and averaged across cells. The results showed that in cells overexpressing tomosyn, this ratio is consistently higher than in controls (Fig. 3e), such that after 45 s of acquisition there is a majority of new vesicles at the membrane (new/old ratio of 1.75±0.1, n = 26 cells) while in control cells, most of the vesicles at the same timepoint are “old” (new/old ratio of 0.72±0.04, n = 18 cells). Indeed, in tomosyn-overexpressing cells, the rate of vesicle arrival and disappearance were significantly higher than in control cells (Table 1), indicating that vesicle turnover at the membrane increases under overexpression of tomosyn.

**Table 1:** Rate of vesicle arrival and disappearance in control and tomosyn overexpressing cells.

	Control	Tomosyn
	(n = 18 cells)	(n = 25 cells)
**Rate of arrival (Vesicles sec^−1^ µm^−2^)**	3.8·10^−3^±0.6·10^−3^	7.3·10^−3^±1.1·10^−3^ *
**Rate of disappearance (Vesicles sec^−1^ µm^−2^)**	1.1·10^−3^±0.3·10^−3^ #	1.9·10^−3^±0.5·10^−3^ #

* p<0.05 Mann Whitney's rank-sum test.

# The rates of disappearance are lower due to the short duration of imaging, since newcomer vesicles often retracted after imaging was completed.

**Figure 4:**
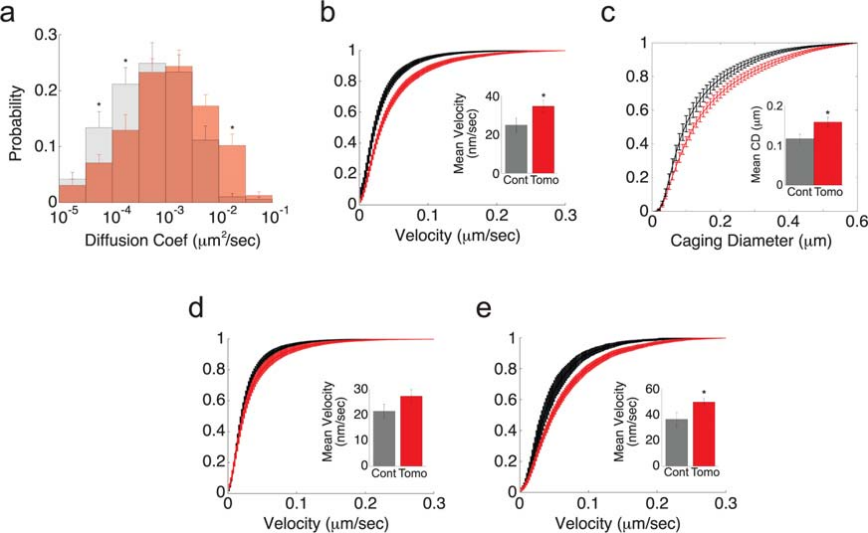
Tomosyn increases the mobility of newcomer vesicles. (a). Logarithmically binned histogram of diffusion coefficients averaged across cells in control (gray) and tomosyn-overexpressing (red) cells. (b). Average mobility cumulative distribution function (cdf) of control (black) and tomosyn-overexpressing (red) cells. Inset shows the mean velocity calculated for control (24.8±3.6 nm s^−1^) and tomosyn-overexpressing cells (34.5±3.1 nm s^−1^). (c). Average cumulative histogram of the caging diameter (CD) of vesicles in control (black) and tomosyn-overexpressing (red) cells. Inset shows the mean CD for tomosyn-overexpressing cells (134±10.3 nm) and controls (112±9.5 nm). (d). Average mobility cdf of resident vesicles in control (black) and tomosyn-overexpressing (red) cells. Inset shows the mean velocity of resident vesicles in control (21.2±2.6 nm s^−1^) and tomosyn-overexpressing cells (27.1±2.4 nm s^−1^). (e). Average ptpv cdf of newcomer vesicles in control (black) and tomosyn-overexpressing (red) cells. Inset shows the mean velocity of newcomer vesicles in control (36.1±5.4 nm s^−1^) and tomosyn-overexpressing cells (49.5±3.0 nm s^−1^). All data presented are from 478 vesicles in 18 control cells and 642 vesicles in 25 tomosyn-overexpressing cells (*p<0.05).

We next measured the mobility of vesicles in control and tomosyn-overexpressing cells. Tomosyn-overexpressing cells showed significantly higher mobility of membrane-proximal vesicles. This was evident from both the mean histogram of diffusion coefficients (Fig. 4a) and the mobility cdf (Fig. 4b). In a recent paper, it was shown that overexpression of Munc13 or application of phorbol 12-myristate 13-acetate (PMA) in bovine chromaffin cells causes a reduction in vesicle mobility [38]. Our results are consistent with these findings, since Munc13 and PMA are known to enhance vesicle priming. These findings provide further support for the hypothesis that tomosyn and Munc13 function as antagonists at the same step in the priming process [21]. Therefore, an increase in vesicle mobility under tomosyn overexpression is expected. To directly compare our results to those of Nofal et al., we measured the caging diameter (CD) of vesicles in control and tomosyn-overexpressing cells. The CD was calculated as the maximal distance that a vesicle travels within a fixed time window of 6 s. In control cells, the average CD was in good agreement with the previous report [38]. Tomosyn overexpression significantly increased the average CD (Fig. 4c), indicating that vesicles in tomosyn-overexpressing cells are less restricted than in control cells.

These results may indicate that the increased mobility of vesicles in tomosyn-overexpressing cells stems from the increased vesicle turnover occurring in these cells. If this is the case, then the mobility of newcomer vesicles, rather than that of resident vesicles, should be the source of the overall changes in mobility between tomosyn and control cells. We therefore compared the mobility of resident and newcomer vesicles between control and tomosyn cells and found that the mobility cdf of resident vesicles in control cells was indistinguishable from that of resident vesicles in tomosyn-overexpressing cells (Fig. 4d). However, the mobility of newcomer vesicles in tomosyn-overexpressing cells was significantly higher than that of newcomer vesicles in control cells (Fig. 4e). This indicates that these vesicles fail to immobilize upon arrival to the membrane-proximal region when tomosyn is overexpressed. We therefore concluded that tomosyn prevents the immobilization of newly arriving vesicles, thereby increasing both vesicle turnover near the membrane and their overall mobility.

### Stimulation of tomosyn-overexpressing cells reveals preferential fusion of newcomer vesicles

**Figure 5:**
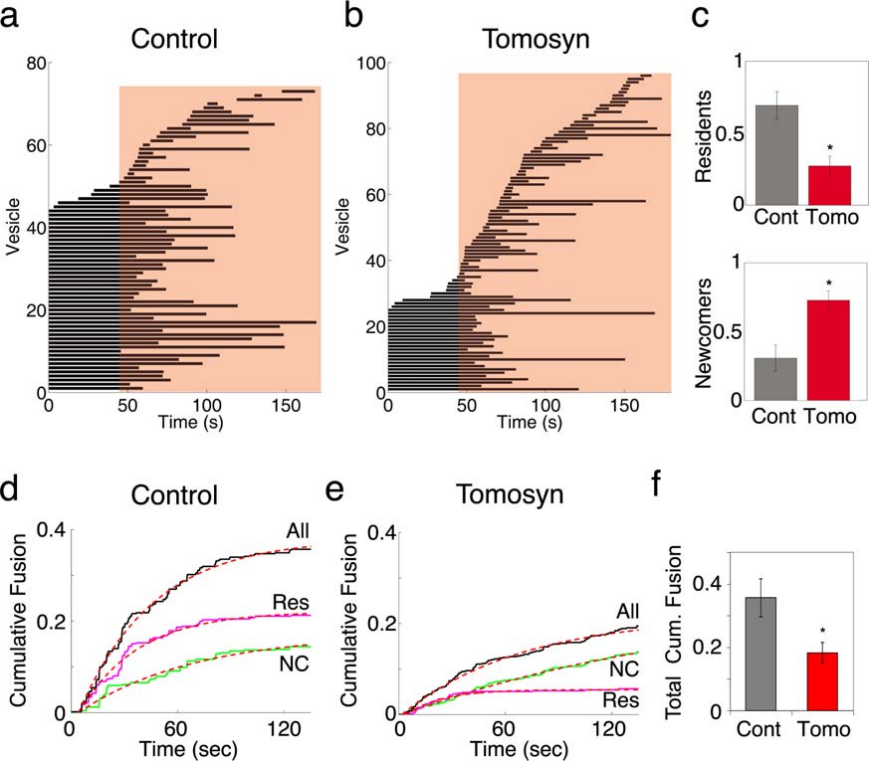
Exocytosis in tomosyn-overexpressing cells occurs mostly from a pool of newcomer vesicles. (a). Kymograph plot for vesicles secreted in control cells (n = 73 vesicles from 9 cells). Stimulation period is indicated by red background. (b). Lifetime plot for vesicles secreted in tomosyn-overexpressing cells (n = 96 vesicles from 16 cells). (c). Average proportion of resident and newcomer vesicles secreted in control and tomosyn-overexpressing cells. (d). Average cumulative fusion plot, normalized to the amount of vesicles visible before stimulation, for control cells. Total secretion, and secretion of resident vesicles and of newcomer vesicles are indicated by black, magenta and green lines, respectively. Dotted red lines represent exponential fits to the data. See Table 2 for rate constants. *t* = *0* is the onset of stimulation. (e). Average cumulative fusion plot, normalized to the amount of vesicles visible before stimulation, for tomosyn-overexpressing cells. Total secretion, secretion of resident vesicles and of newcomer vesicles are indicated by black, magenta and green lines, respectively. Dotted red lines represent exponential fits to the data. See Table 2 for rate constants. *t* = *0* is the onset of stimulation. (f). Average total normalized secretion in control and tomosyn-overexpressing cells, expressed as the fraction of the amount of vesicles in the TIRF plane prior to stimulation.

Electrophysiological measurements have shown that tomosyn causes a reduction in the number of primed vesicles and as a result, a reduction in the fusion of vesicles from the RRP [18]. Here we show that fusion in control cells occurs mainly from a pool of resident vesicles, which have low mobility. Thus, we wanted to examine whether the reduction in the amount of fusion-competent vesicles in tomosyn-overexpressing cells is related to changes in the characteristics of pre-fusion mobility and residence time of individual vesicles as viewed with TIRF. To monitor fusion events occurring in control and tomosyn-overexpressing cells, we took advantage of the inherent pH sensitivity of the Venus fluorophore. Venus, unlike mRFP, responds to neutral pH with a brightening of its fluorescence, resulting in a fluorescent flash that appears each time a vesicle fuses, exposing its lumen to the extracellular solution [50]. We therefore used this property to detect fusion events in cells infected with the viruses described in Figure 3a. The cells were stimulated according to the protocol described in Figure 2. We then pooled together all of the vesicles that underwent exocytosis in control and tomosyn-overexpressing cells. To obtain similar amounts of fusion events, we recorded exocytosis from 16 tomosyn-overexpressing cells (Fig. 5b; n = 96 vesicles) compared to 9 control cells (Fig. 5a; n = 73 vesicles). In line with the inhibitory role of tomosyn, overall secretion, normalized to the amount of vesicles visible in each cell's footprint before stimulation, was reduced in tomosyn-overexpressing cells compared to controls (Fig. 5f, Table 2). It is also clear from Figures 5a and b that whereas control cells secreted mostly resident vesicles (Fig. 5c, residents), in tomosyn-overexpressing cells, most of the secreted vesicles arrived during stimulation and fused shortly thereafter (Fig. 5c, newcomers) while secretion from the resident pool was inhibited (Fig. 5c, residents).

**Table 2:** Fusion of resident and newcomer vesicles in control and tomosyn overexpressing cells.

		Control	Tomosyn
		(n = 9 cells)	(n = 16 cells)
**All Vesicles**	**% Fusion**	35.6±6%	18.3±2.5%
	**Rate constant**	42 s	68 s
**Resident Vesicles**	**% Residents at stimulus onset**	71±4.1%	60.8±4.3%
	**% of Residents that fused**	28.6±3.2%	9.1±2.8% *
	**Rate constant**	33.3 s	20.3 s
**Newcomer Vesicles**	**Arrival rate of Newcomers (Ves µm^−2^ s^−1^)**	3.4·10^−3^±7.6·10^−4^	5.9·10^−3^±1.9·10^−3^
	**% of Newcomers that fused**	4.2±1.1%	3.6±0.6%
	**Rate constant**	65.5 s	177.8 s †

* p<0.005 student's t-test.

† Estimated time constant.

The normalized cumulative fusion trace shows that the kinetics of secretion were also altered in tomosyn-overexpressing cells (Fig. 5d,e, black traces). Exponential fitting of the cumulative secretion curves (Fig. 5d,e, red dotted lines) showed that secretion in tomosyn-overexpressing cells is significantly slower than in controls (Fig. 5e and 5d, black traces; Table 2). This is consistent with previous work on tomosyn [18], [26], [28] and in agreement with a recent publication showing that the kinetics of exocytosis as observed with TIRF is comparable to global secretion as observed with electrophysiological methods [50]. Measuring the time constants of secretion of the two vesicle populations (residents and newcomers) showed that the fusion of resident vesicles occurs at a similar rate in both control and tomosyn-overexpressing cells (Fig. 5d and 5e, magenta traces; Table 2). However, the proportion of resident vesicles that fused in tomosyn-overexpressing cells was significantly lower than in control cells (Table 2). These data suggest that tomosyn reduces the release probability of resident vesicles.

**Figure 6:**
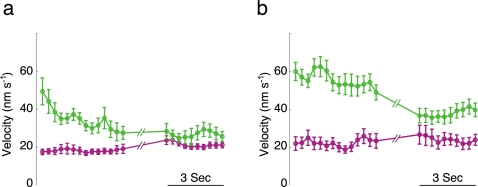
Tomosyn delays the immobilization of newcomer vesicles before fusion. (a). Point-to-point velocity (ptpv) of newcomer (green) and resident (magenta) vesicles that fused in response to stimulation in control cells. The graph depicts the ptpv of vesicles during the first 4 s of their arrival near the membrane and then during the last 3 s before fusion. The ptpv of resident vesicles is shown for the first 4 s of acquisition and the last 3 s before fusion (magenta). (b). As in a, ptpv for resident and newcomer vesicles in tomosyn-overexpressing cells.

The kinetics of sustained secretion in tomosyn cells was dictated by the fusion of newcomer vesicles (Fig. 5e, green curve). Although the arrival rate of newcomers in tomosyn cells was higher compared to control cells, the release rate of these vesicles was lower in tomosyn cells (Table 2). Assuming that immobilization is a required step in the process of vesicle priming, we hypothesized that tomosyn reduces the release rate of newcomer vesicles by delaying their immobilization. We therefore measured the mobility of newcomer vesicles that fuse upon stimulation, from their arrival in the TIRF plane up until their fusion. This analysis showed that vesicles in control cells are immobilized within seconds of their arrival, reaching a low-mobility state that is similar to their mobility during the last 3 s before fusion (Fig. 6a, green curve). In contrast, newcomer vesicles that fused in tomosyn-overexpressing cells failed to immobilize upon arrival and reached their lowest mobility only prior to fusion. Their mobility in this low-mobility state, however, was still higher than that of their control counterparts (compare Fig. 6b, to Fig. 6a; green curves). Resident vesicles in both control and tomosyn-overexpressing cells were in a low-mobility state during the entire time of acquisition, indicating that, although it reduces their fusion (Fig. 5d and 5e, Table 2), tomosyn has no effect on the mobility of resident vesicles once they are immobilized (Fig. 6a,b, magenta curves). Taken together, these findings indicated that tomosyn inhibits exocytosis by both attenuating vesicle immobilization and thereby delaying the fusion of newcomer vesicles, and decreasing the probability of release of resident vesicles.

## Discussion

The aim of this study was to examine the physical manifestation of vesicle priming and understand how tomosyn, a specific vesicle priming inhibitor [21], [24], [29], [30], [49] regulates vesicle dynamics. Our initial finding was that tomosyn causes an increase in the lateral mobility of vesicles. This shift in vesicle mobility resulted specifically from the enhanced mobility of newcomer vesicles, indicating that tomosyn prevents these vesicles from immobilizing. Further support for this conclusion came from the finding that tomosyn attenuates the immobilization of newcomer vesicles prior to fusion. This result, together with the finding that the mobility of newcomer vesicles before fusion is higher in tomosyn cells than in control cells may be the reason for the slower release kinetics of these vesicles. Taken together, these findings suggest that tomosyn regulates vesicle immobilization, indicating that immobilization is a prerequisite for entry into the primed state. We further showed that tomosyn exerts a negative effect on the fusion probability of resident, low-mobility vesicles. This suggested that immobilization is not sufficient for fusion competence and further molecular processes, in which tomosyn is involved, occur following immobilization.

We also demonstrate that tomosyn reduces the residence time of vesicles in the membrane-proximal region, leading to increased vesicle turnover at the plasma membrane. These results are consistent with the reduced membrane-residence time of vesicles after cleavage of the SNARE proteins [33]. A reduction in vesicle residence time was also observed in chromaffin cells from Munc18-knockout mice [37]. Interestingly, tomosyn and Munc18 both compete for binding to Syntaxin [22], [25]. However, while Munc18 has an enhancing effect on secretion [51], [52], tomosyn plays an inhibitory role. The similarities between these effects point to a common mechanism that involves modulation of membrane-bound Syntaxin. This is supported by the finding that Syntaxin cleavage causes a significant decrease in the number of docked vesicles in chromaffin cells [39] and in *Caenorhabditis elegans* [40].

The effect of tomosyn on both newcomer and resident vesicle populations can be explained by a simple mechanism. We can speculate that resident, low-mobility vesicles are tethered by the formation of SNARE complexes between the vesicle and membrane [38]–[40]. However, while immobilization could result from as few as one trans-SNARE complex, multiple SNARE complexes would have to form on a single vesicle in order to render it fusion-competent [53], [54]. The effect of tomosyn on the immobilization of newcomer vesicles may be mediated by a general reduction in the ability to form trans-SNARE complexes. This would affect newly arriving vesicles, causing a reduction in the capacity to form the first trans-SNARE complex and would therefore lead to an increase in vesicle turnover. Tomosyn's effect on resident vesicles may be explained by a similar mechanism: i.e. tomosyn attenuates the formation of the subsequent *trans*-SNARE complexes hence reducing the number of SNARE complexes per vesicle, which may lead to a reduction in the release of resident vesicles [53], [54]. In such a situation, the vesicle could still be docked [39], [40] and restricted in its mobility (having the first SNARE complex formed), but the probability of its release would remain low. Tomosyn may affect syntaxin directly via its SNARE motif [26], [29], [55] or indirectly through interaction of the SNARE proteins with its N-terminal domain that is predicted to fold into a beta-propeller-like structure as was recently shown for Sro7, the yeast tomosyn homologue [23]. However, further experiments are needed to understand the dynamic interaction between the mammalian tomosyn and the SNARE proteins during exocytosis.

A profound effect of tomosyn was a decrease in the fusion of resident vesicles and an increase in the contribution of newcomer vesicles to the exocytotic response. In neurons, overexpression of tomosyn phospho-mutants causes a decrease in synchronous release and an increase in asynchronous release [19], indicating that inhibition of priming by tomosyn shifts the kinetics of release, favoring late asynchronous release. Similar effects have been observed in *C. elegans* Munc13-knockout animals, where the severe priming defect was rescued by the open form of Syntaxin [56]. Synaptic transmission under these conditions was partially restored but was slower and shifted from synchronous to asynchronous release, similar to the effect of tomosyn. The increased fusion of newcomer vesicles in our experiments may reflect a similar situation, given that the fusion of resident vesicles can be more tightly coupled to stimulation than the fusion of newcomers. Taken together, these data suggest that inhibition of priming is associated with a change in fusion pattern from synchronous to asynchronous release.

Previous work on vesicle priming has demonstrated that primed vesicles cannot be distinguished from morphologically docked vesicles by electron microscopy [31]. This implies that functional docking and entry into the primed state may significantly differ from structural docking. Consistent with this, and despite the significant impairment in exocytosis under tomosyn overexpression, the number of visible, docked vesicles in the TIRF plane is not altered (as also evidenced by electron microscopy [18]). Nevertheless, tomosyn increases the vesicle turnover rate and most of the fusion events during stimulation originate from a pool of newcomer vesicles. Therefore, although tomosyn did not alter the steady-state number of vesicles at the membrane, our data indicate that tomosyn has a significant effect on docking and undocking kinetics. It is possible that when SNARE-complex formation is blocked, vesicles undergo undocking more frequently as other docking machineries are less effective at retaining the vesicles near the membrane. This is supported by recent findings showing that cleavage of syntaxin in chromaffin cells causes a loss of morphologically docked vesicles, observed with cryo-electron microscopy [39]. Moreover, in *C. elegans*, deletion of tomosyn causes enhanced morphological docking, perhaps due to an increase in vesicle immobilization at the plasma membrane through SNARE-complex formation [30].

To conclude, we show here that tomosyn modulates vesicle priming by preventing the immobilization of vesicles at the membrane. During stimulation, tomosyn causes the preferential release of newcomers over resident vesicles and attenuates immobilization of the former, resulting in a reduction in the rate of vesicle fusion. Our results further indicate that immobilization is necessary but not sufficient to achieve a fusion-competent state. Although it is still well accepted that docking and priming are two distinct, sequential steps mediated by almost completely separate molecular mechanisms, our findings, together with recent studies [37], [38], suggest that these two steps are interlinked molecularly Therefore, interfering with the priming process attenuates the fusion of vesicles as predicted, but changes also the dynamics of vesicle docking. The emerging definition of “functional docking” therefore constitutes the first step in vesicle priming, and it is followed by molecular modifications that do not translate into changes in vesicle mobility.

## Materials and Methods

### Plasmids, chromaffin cell preparation and transfection

pSFV1-IRES-NPY-Venus plasmid was a kind gift of Ulf Matti (Saarland University, Homburg, Germany). Rat m-tomosyn cDNA was cloned into the BamHI site of this plasmid, located upstream of the IRES element, and its sequence was confirmed by automated sequencing. Virus particles were prepared as previously described [57]. Overexpression of tomosyn using this system has previously been determined to be ∼13-fold over endogenous tomosyn (Yizhar et al. 2004). Isolated bovine adrenal chromaffin cells were prepared and cultured as described previously [18], [57]. Cultured cells were infected 5–48 h after plating [57] and used for imaging 8–12 h later. For dual-wavelength imaging, cells were electroporated immediately after culturing with 40 µg DNA containing equal quantities of NPY-mRFP and SpH plasmids. After 24 h, the cells were re-plated on glass coverslips coated with poly-D-lysine (Sigma) and imaged the following day. Control and tomosyn-overexpressing cells were always imaged on the same day and after an identical infection time.

### Evanescent wave imaging

Imaging was carried out with an Olympus IX-70 inverted microscope with a 60× (TIRF) objective (Olympus) and a T.I.L.L photonics TIRF condenser (T.I.L.L photonics, Gräfelfing, Germany). Laser excitation was provided by two solid-state lasers (Laser Quantum, Stockport, UK) emitting at 473 nm and 532 nm. The decay constant for the evanescent field was calculated according to [58] and was determined to be 141 nm. An Andor Ixon 887 EMCCD camera (Andor, Belfast, Northern Ireland) was used to acquire images, controlled by Metamorph software (Molecular Devices, Downingtown, PA). Dual-wavelength imaging was carried out using a Dual-View beam-splitter device from Optical Insights (Roper Bioscience, Tuscon, AZ). Time-lapse images were acquired at frame rate and the acquisition rate was 5 Hz for single-wavelength and 3.3 Hz for dual-wavelength imaging. The microscope was enclosed in a custom-built temperature-controlled acrylic-glass cage that was set to 32°C, both to provide the cells with adequate temperature and to minimize focus drift. Cells were constantly perfused with solution containing (in mM): 140 NaCl, 3 KCl, 2 CaCl_2_, 1 MgCl_2_, 10 HEPES and 2 mg/ml glucose pH 7.2 (320 mOsm). Cells were depolarized by application of a similar solution in which 60 mM NaCl was replaced with KCl.

### Image processing and data analysis

Time-lapse images of NPY-Venus or NPY-mRFP fluorescence were pre-processed by subtracting from each image a low-passed version of itself (1/µm spatial frequency) and smoothing the resulting image by low-pass filtering with spatial frequency of 0.2/µm. Vesicles were identified as diffraction-limited objects that were significantly brighter than the set fluorescence threshold. Tracking was performed by fitting each vesicle with a 2D Gaussian distribution to precisely identify the vesicle's coordinates (see Figure S1). Vesicles that appeared for less than 2 s were not considered for the analysis, and trajectories of vesicles that collided were only used until the time of collision. In addition, calculation of vesicle mobility before fusion showed that there was a slight increase in vesicle mobility during the last 600 ms before fusion, as reported previously [32], [59]. Since the source of these movements is unclear and may be related to the fusion process itself or to the activity of molecular motors [59], [60], we omitted these points from the analysis (Fig. 6).

The apparent diffusion coefficient was calculated as previously described [34]. CD was calculated as described in Nofal et al. [38]. The windowed velocity calculation was performed by calculating the mean ptpv of the vesicle within a running window of 1 s. This calculation measures the short-range jittering motions of the vesicles and approximates the initial phase of the mean squared displacement curve [44]. Statistical analysis showed that the cdfs of the CD and windowed velocity of individual vesicles were distributed log-normally (see Figure S2 and Analysis S1). For each cell, the mean cdf was calculated from the individual vesicle cdfs and then averaged across cells to compare between experimental conditions.

SpH time-lapse sequences were processed in the following manner: each frame in the stimulation sequence was divided by an averaged image of the pre-stimulus background fluorescence. Fusion events were detected as bright spots that transiently appeared and then dimmed by lateral dispersion that was associated with disappearance of NPY-mRFP fluorescence at the same location in the red channel. Since the number of fusing vesicles in each cell was relatively small (8.1±2.2 events/cell, constituting 27±5% of the number of vesicles visible in the TIRF plane before stimulation), we pooled fusion events from several cells to further analyze their characteristics. For each cell, a cumulative fusion vector was constructed, such that each fusion event contributed 1 unit at the time of its occurrence. This trace was then normalized to the number of vesicles visible in the pre-stimulation period, such that each point indicated the fraction of vesicles released. Tracking and data analysis were performed using custom-written Matlab programs (Mathworks, Natick, MA). Error bars in all figures represent SEM.

## Supporting Information

Analysis S1 Detailed methods and statistical analysis.
(0.04 MB DOC)

Figure S1 Vesicle-tracking algorithm (a). Image showing a typical chromaffin cell with vesicles marked by infection with pSFV1-IRES-Venus-NPY. Enlarged region (top right) shows a representative vesicle and the graph (bottom right) shows a 3D representation of the vesicle-intensity distribution. To prevent tracking error due to close vesicle proximity, we used the following procedure: during subsequent frames, a 3×3 matrix was constructed around the previous location of the vesicle (b, red lines and green crosses, respectively), with each square about the size of one vesicle (250 nm). The fitting procedure was attempted in each of the squares and if objects were found in more than one square, their properties (location, intensity, half-width, derived from the 2D Gaussian fit) were compared to those of the vesicle from the previous frame. The best match was designated as the same vesicle from the previous image and its position was recorded in the calculated trajectory (b, blue crosses). This procedure enabled high-precision tracking, even when two vesicles were only pixels apart. The procedure was repeated for each vesicle in each frame and vesicle trajectories were thus collected for analysis.
(10.71 MB TIF)

Figure S2 Statistical analysis of mobility distributions (a). Windowed velocity calculation of a single vesicle from a control cell, as detailed in Figure 1. (b). Mean histogram of velocity values for 35 vesicles from a single control cell. (c). Mean histogram of the logarithms of velocity values for the same 35 vesicles as in b. Dashed red line represents fitting to a normal distribution model. (d). Cumulative distribution function of velocity values for the vesicle shown in a (black), fitted with a log-normal distribution model (dashed red line).
(11.56 MB TIF)

Figure S3 Mobility correction with fixed beads (a). Mobility cdf of fixed 220-nm beads imaged at 10 Hz at varying laser intensities (each line represents the mean cdf of a group of beads imaged at a specific laser intensity as shown in the legend; n = 31 beads in each condition). (b). The mean apparent mobility of each bead was plotted against its fluorescence intensity (gray dots). Red line represents curve-fitting to the data as detailed in the Supplementary Methods. (c) Mobility cdfs calculated for the same bead groups shown in a, after correction for noise-related mobility. Note that apparent mobility is strongly reduced and the differences between the apparent mobilities of the beads under different illumination intensities are significantly smaller. (d). Mean mobility values of vesicles from all control cells measured in our experiments, plotted against their mean fluorescence intensity before (black dots), and after (red dots) correction. Note that vesicle fluorescence was relatively strong, such that the maximal mobility artifact before correction is on the order of 20 nm s−1.
(11.06 MB TIF)
