# Supplementary material for: Modulating Vesicle Priming Reveals that Vesicle Immobilization Is Necessary but not Sufficient for Fusion-Competence
Source: PLoS One. 2008 Jul 16;3(7):e2694. doi: 10.1371/journal.pone.0002694 (PMC2444019; doi:10.1371/journal.pone.0002694)
Supplement: Analysis S1 — Detailed methods and statistical analysis. (0.04 MB DOC) [file pone.0002694.s001.doc]

**Supplementary Information**

**Tracking procedure for analysis of vesicle mobility**

We employed a Gaussian-fitting algorithm [1] modified to perform under high vesicle density, where two vesicles may be confused by ordinary tracking, thereby introducing tracking error (summarized in Figure S1). Briefly, vesicles were identified according to their distinct point-spread function fluorescence. This fluorescence distribution was used to fit a 2D Gaussian function to precisely localize the peak of fluorescence which corresponds to the center of the vesicle:

$$G(x,y)=A\cdot\exp\left[-\frac{(x-x_{0})^{2}+(y-y_{0})^{2}}{B}\right]$$

where (*x_0_ ,y_0_*) are the central coordinates of the vesicle and A and B are parameters describing the distribution. The tracking procedure was implemented in Matlab and used throughout this study. Automated tracking was performed as described in Figure S1 on a high-pass filtered version of the image sequence. After automated tracking was completed, individual trajectories were manually reviewed to ensure that no tracking errors had occurred. Actual vesicle intensities were then calculated from the original image by measuring the fluorescence of a circular region with a diameter of 300 nm, centered around the *(x,y)* coordinates derived from the tracking algorithm and subtracting the mean fluorescence of a 160-nm-wide annulus surrounding this region.

**Statistical analysis of mobility and caging diameter (CD) cumulative distribution functions (cdfs)**

To optimally represent the mobility of individual vesicles, we calculated a windowed velocity graph for each, as detailed in the text, and a mobility cdf as shown in Figure S2a. These distributions were skewed so that lower mobility values predominated (Figure S2b). However, the logarithm of the data distributed normally (Figure S2c), indicating that these data may distribute log-normally. We therefore calculated a mean mobility cdf for each cell included in our mobility experiments (n = 43 cells) and performed the Lilliefors test on the logarithm of each distribution. This test confirmed that the logarithms of the values were normally distributed (*p* < 0.05 in all 43 cells), indicating that the original mobility values indeed distribute log-normally. This indicates that calculating an arithmetic mean (as when calculating the mean squared displacement—MSD) is not an appropriate method to assess the degree of vesicle mobility, since the movements of vesicles on a short time scale do not distribute normally. It was therefore important to find the parameters that would accurately portray the vesicles’ mobility.

We therefore fitted the mobility cdf of each vesicle with the log-normal cumulative probability density function:

$$p=F(\left.x\right|\mu,\sigma)=\frac{1}{\sigma\sqrt{2\pi}}\int_{0}^{x} \frac{e^{\frac{-(\ln(x)-\mu)^{2}}{2\sigma^{2}}}}{x}dx$$

where μ and σ are the mean and standard deviation, respectively, of the associated normal distribution (Figure S2d, dashed red line). The μ parameter was used to describe the vesicle’s representative mobility and is therefore referred to as the “mean mobility” of the vesicle throughout the manuscript. We also found that the CD values for individual vesicles distributed in a similar manner (not shown), and therefore the cumulative-probability histograms of vesicle CDs were fitted with the same function, and the μ parameter of each vesicle’s CD cdf was used to represent the vesicle’s caging behavior.

**Correction of vesicle mobility values for bias introduced by instrumental noise**

In single-particle tracking, the calculated mobility is influenced by the signal-to-noise ratio, which deteriorates with the fluorescence of the particle. To correct for this inherent bias, we performed single-particle tracking of 220-nm beads that were fixed to the glass coverslip and calculated their apparent mobility at varying fluorescence intensities (Figure S3a). Note that the mobility artifact is stronger as laser intensity declines (e.g. 45% versus 95% of maximal power), demonstrating the contribution of instrument noise to the mobility calculation [2]. The mean mobility was plotted against fluorescence intensity for each bead (Figure S3b) and the scatter was fitted by the function: $V=A\times x^{b}$where V is the velocity artifact and A and b are parameters describing the dependence of the mobility artifact on fluorescence intensity. Fitting results were: A = 0.24, b *=* -0.476 (Figure S3b, red line). Mobility values calculated for each vesicle throughout this study were then corrected by subtracting the artifactual mobility resulting from instrument noise along each point in the trajectory according to the vesicle’s intensity at that point (Figure S3c).

**References:**

1. Cheezum, M.K., W.F. Walker, and W.H. Guilford, *Quantitative comparison of algorithms for tracking single fluorescent particles.* Biophys J, 2001. 81(4): p. 2378-88.

2. Allersma, M.W., et al., *Motion matters: secretory granule motion adjacent to the plasma membrane and exocytosis.* Mol Biol Cell, 2006. 17(5): p. 2424-38.

**Supporting Figure Legends**

**Figure S1. Vesicle-tracking algorithm**

**(a).** Image showing a typical chromaffin cell with vesicles marked by infection with pSFV1-IRES-Venus-NPY. Enlarged region (top right) shows a representative vesicle and the graph (bottom right) shows a 3D representation of the vesicle-intensity distribution. To prevent tracking error due to close vesicle proximity, we used the following procedure: during subsequent frames, a 3 x 3 matrix was constructed around the previous location of the vesicle (**b**, red lines and green crosses, respectively), with each square about the size of one vesicle (250 nm). The fitting procedure was attempted in each of the squares and if objects were found in more than one square, their properties (location, intensity, half-width, derived from the 2D Gaussian fit) were compared to those of the vesicle from the previous frame. The best match was designated as the same vesicle from the previous image and its position was recorded in the calculated trajectory (**b**, blue crosses). This procedure enabled high-precision tracking, even when two vesicles were only pixels apart. The procedure was repeated for each vesicle in each frame and vesicle trajectories were thus collected for analysis.

**Figure S2. Statistical analysis of mobility distributions**

**(a)**. Windowed velocity calculation of a single vesicle from a control cell, as detailed in Figure 1. **(b)**. Mean histogram of velocity values for 35 vesicles from a single control cell. **(c)**. Mean histogram of the **logarithms** of velocity values for the same 35 vesicles as in **b**. Dashed red line represents fitting to a normal distribution model. **(d).** Cumulative distribution function of velocity values for the vesicle shown in **a** (black), fitted with a log-normal distribution model (dashed red line).

**Figure S3. Mobility correction with fixed beads**

**(a).** Mobility cdf of fixed 220-nm beads imaged at 10 Hz at varying laser intensities (each line represents the mean cdf of a group of beads imaged at a specific laser intensity as shown in the legend; n = 31 beads in each condition). **(b).** The mean apparent mobility of each bead was plotted against its fluorescence intensity (gray dots). Red line represents curve-fitting to the data as detailed in the Supplementary Methods. **(c)** Mobility cdfs calculated for the same bead groups shown in **a**, after correction for noise-related mobility. Note that apparent mobility is strongly reduced and the differences between the apparent mobilities of the beads under different illumination intensities are significantly smaller. **(d).** Mean mobility values of vesicles from all control cells measured in our experiments, plotted against their mean fluorescence intensity before (black dots), and after (red dots) correction. Note that vesicle fluorescence was relatively strong, such that the maximal mobility artifact *before correction* is on the order of 20 nm s^-1^.
