# Supplementary figures and images for: Modulating Vesicle Priming Reveals that Vesicle Immobilization Is Necessary but not Sufficient for Fusion-Competence
Source: PLoS One. 2008 Jul 16;3(7):e2694. doi: 10.1371/journal.pone.0002694 (PMC2444019; doi:10.1371/journal.pone.0002694)

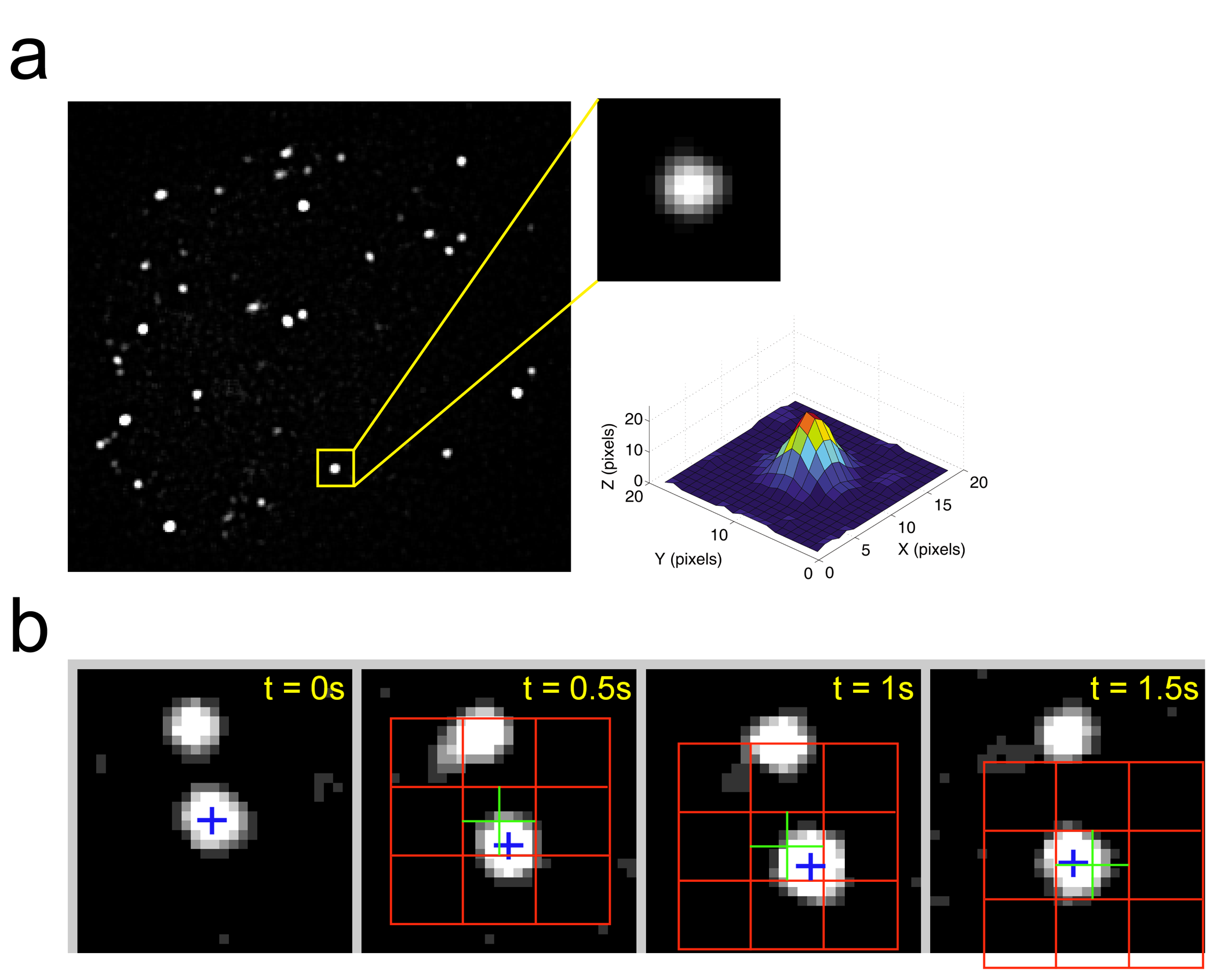

Supplement: Figure S1 — Vesicle-tracking algorithm (a). Image showing a typical chromaffin cell with vesicles marked by infection with pSFV1-IRES-Venus-NPY. Enlarged region (top right) shows a representative vesicle and the graph (bottom right) shows a 3D representation of the vesicle-intensity distribution. To prevent tracking error due to close vesicle proximity, we used the following procedure: during subsequent frames, a 3×3 matrix was constructed around the previous location of the vesicle (b, red lines and green crosses, respectively), with each square about the size of one vesicle (250 nm). The fitting procedure was attempted in each of the squares and if objects were found in more than one square, their properties (location, intensity, half-width, derived from the 2D Gaussian fit) were compared to those of the vesicle from the previous frame. The best match was designated as the same vesicle from the previous image and its position was recorded in the calculated trajectory (b, blue crosses). This procedure enabled high-precision tracking, even when two vesicles were only pixels apart. The procedure was repeated for each vesicle in each frame and vesicle trajectories were thus collected for analysis. (10.71 MB TIF) [file pone.0002694.s002.tif]

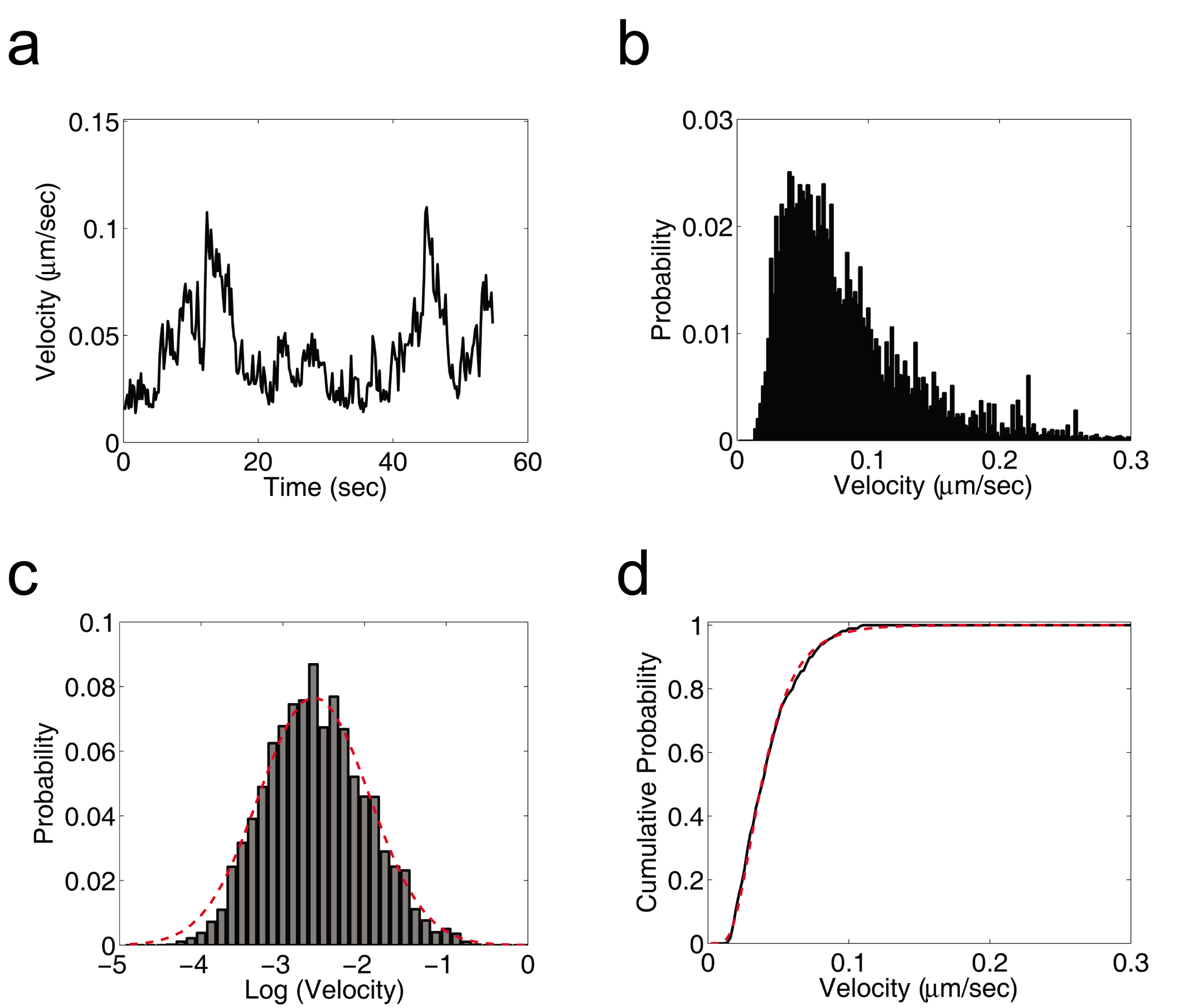

Supplement: Figure S2 — Statistical analysis of mobility distributions (a). Windowed velocity calculation of a single vesicle from a control cell, as detailed in Figure 1. (b). Mean histogram of velocity values for 35 vesicles from a single control cell. (c). Mean histogram of the logarithms of velocity values for the same 35 vesicles as in b. Dashed red line represents fitting to a normal distribution model. (d). Cumulative distribution function of velocity values for the vesicle shown in a (black), fitted with a log-normal distribution model (dashed red line). (11.56 MB TIF) [file pone.0002694.s003.tif]

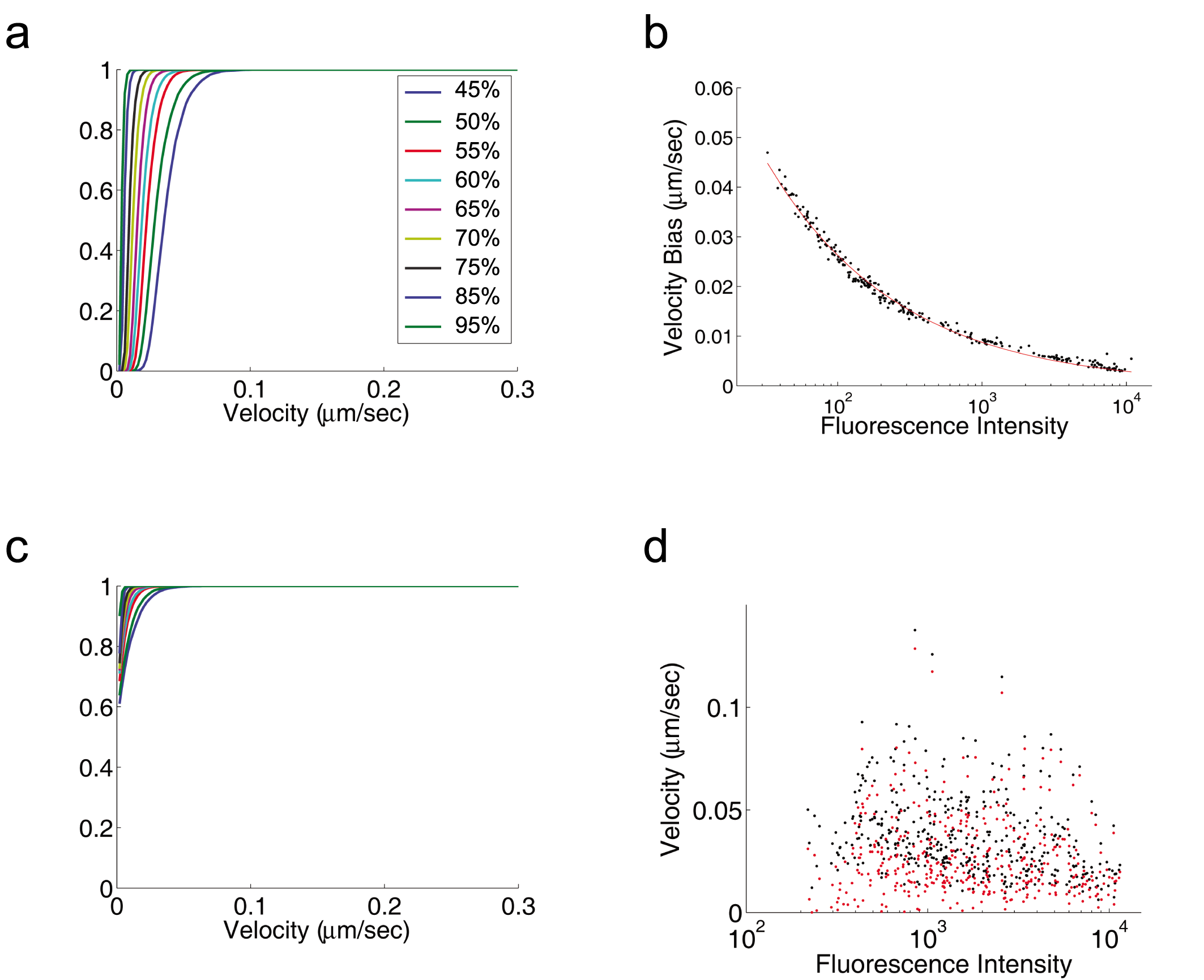

Supplement: Figure S3 — Mobility correction with fixed beads (a). Mobility cdf of fixed 220-nm beads imaged at 10 Hz at varying laser intensities (each line represents the mean cdf of a group of beads imaged at a specific laser intensity as shown in the legend; n = 31 beads in each condition). (b). The mean apparent mobility of each bead was plotted against its fluorescence intensity (gray dots). Red line represents curve-fitting to the data as detailed in the Supplementary Methods. (c) Mobility cdfs calculated for the same bead groups shown in a, after correction for noise-related mobility. Note that apparent mobility is strongly reduced and the differences between the apparent mobilities of the beads under different illumination intensities are significantly smaller. (d). Mean mobility values of vesicles from all control cells measured in our experiments, plotted against their mean fluorescence intensity before (black dots), and after (red dots) correction. Note that vesicle fluorescence was relatively strong, such that the maximal mobility artifact before correction is on the order of 20 nm s−1. (11.06 MB TIF) [file pone.0002694.s004.tif]
